# From health-giving to disease-ridden: the Tietê river from the perspective of São Paulo’s rowing clubs, 1900-1940

**DOI:** 10.1590/S0104-59702023000100035

**Published:** 2023-08-11

**Authors:** Daniele Cristina Carqueijeiro de Medeiros, Marcelo Moraes e Silva, Evelise Amgarten Quitzau

**Affiliations:** i Professora, Instituto Superior de Educación Física/Universidad de la República. Paysandú – Uruguay dmedeiros@cup.edu.uy; ii Professor, Departamento de Educação Física/Universidade Federal do Paraná.Curitiba – PR – Brasil marcelomoraes@ufpr.br; iii Professora, Faculdade de Educação Física/Universidade Federal de Viçosa. Viçosa – MG – Brasil evelise.quitzau@ufv.br

**Keywords:** History of health, History of sport, Tietê river, Water sports, História da saúde, História do esporte, Rio Tietê, Esportes aquáticos

## Abstract

This article analyzes changing conceptions of the Tietê river, in São Paulo, Brazil, in the first four decades of the twentieth century as perceived by rowing clubs and the sports press. The historical sources consulted were local newspapers and magazines produced by the clubs. Between 1900 and 1920, as these institutions started to offer water sports, the discourse in the sources vis-a-vis the promotion of health through such sports is positive. However, this relationship changes in the 1930s and 1940s. The Tietê, once synonymous with sport, health, and entertainment, becomes so polluted that it is considered inadequate, making sporting events on its waters unfeasible.

## Resumo

O artigo analisa concepções veiculadas pelos clubes de remo e imprensa esportiva sobre o rio Tietê nas primeiras quatro décadas do século XX, em São Paulo. As fontes históricas utilizadas foram jornais paulistanos e revistas produzidas pelos clubes. Entre 1900 e 1920, tais instituições deram início a práticas esportivas aquáticas, e as fontes apontam um discurso positivo veiculado à promoção da saúde pelos esportes. Entretanto, essa relação se alterou nas décadas de 1930 e 1940. De espaço indissociável das práticas esportivas, da saúde e dos divertimentos, o Tietê passou a ser considerado inadequado, dada a poluição do rio e a impossibilidade de realização de provas esportivas.

O rio Tietê é o principal rio que corta a cidade de São Paulo. Ainda no tempo em que São Paulo era um vilarejo, ele serviu para o transporte de pessoas e para prover alimentos. A partir do momento em que a cidade começou a se urbanizar, seu leito forneceu areia e pedra para a construção dos prédios. Já no século XX, em seus afluentes foram instaladas hidroelétricas responsáveis por abastecer toda a região com energia elétrica (Nóbrega, 1978; Jorge, 2006; Zanirato, 2011).

Conforme salienta Sevcenko (2000, p.28), as várzeas mais distantes do rio Tietê, em conjunto com as dos rios Pinheiros e Tamanduateí, conformavam uma "moldura de águas lodosas, ponteada por casebres humildes por toda a extensão". As características naturais da região, aliadas às intervenções realizadas pela empresa de energia Light, faziam com que as margens do Tietê se tornassem locais de constantes inundações, que atingiam gravemente as populações que habitavam seus arredores, formadas em grande parte por imigrantes de origem europeia, que, paulatinamente, passaram a conviver com negros e mestiços que também habitavam essas áreas (p.30). Essas constantes inundações, além de representar um importante problema para as populações estabelecidas às margens do rio, também passariam a ser vistas pelos clubes e periódicos como um problema da cidade durante as primeiras décadas do século XX.

Para além de seus usos mais objetivos, tanto no que diz respeito às atividades econômicas como ao estabelecimento de moradias em suas margens, o rio também foi um espaço de sociabilidade dos habitantes da cidade. Populações ribeirinhas acampavam em suas margens para pescarias; jovens desafiavam a correnteza das águas para mergulhar; homens e mulheres passeavam em suas pontes para ver o pôr do sol (Bruno, 1954). A partir de 1899, foram encontrados indícios da promoção de novos divertimentos em suas águas: os esportes aquáticos. A natação e o remo começaram a se institucionalizar a partir da instalação em suas margens de alguns clubes de regatas (Nicolini, 2001; Almeida et al., 2013; Medeiros, 2020, 2021, 2022).

De acordo com Jorge (2006) e Sant'Anna (2007), entre 1890 e 1940 houve um profícuo relacionamento entre os moradores de São Paulo e o Tietê. O rio acolhia diversas práticas de diferentes dimensões da vida social; ao mesmo tempo, fornecia recursos naturais imprescindíveis ao processo de urbanização. Essa intensa relação começou a ser desconstruída nos anos 1940, quando a poluição das vias aquáticas e a implantação de rodovias em suas margens impediram que os moradores da cidade usufruíssem das águas.

Assim, o objetivo do presente artigo é analisar a forma como os discursos proferidos a respeito do rio, especialmente pela imprensa e pelos clubes esportivos, alteraram-se ao longo das primeiras quatro décadas do século XX. De espaço adequado às práticas esportivas e ligado à promoção da saúde, o rio passou a ser associado a doenças e contaminação. Esses discursos tiveram dois vieses. O primeiro foi uma concatenação dos rios aos divertimentos saudáveis e ao fortalecimento do corpo, especialmente quando esportes como remo e natação começaram a ser praticados em suas águas. O segundo foi uma mudança de interpretação, quando os clubes deixaram de fomentar esportes nos rios e começaram a promovê-los em espaços como piscinas e represas. A partir desse momento, o Tietê se tornou sinônimo de transtornos, sujeira e contaminação.

A documentação utilizada para esta pesquisa foi formada por jornais paulistanos (*Correio Paulistano, A Gazeta*), encontrados na Hemeroteca Digital da Fundação Biblioteca

Nacional, e revistas produzidas pelos Clube Esperia e Clube de Regatas Tietê, localizadas no acervo dessas instituições esportivas. O jornal *Correio Paulistano* conta com mais de 15.175 edições na Hemeroteca Digital no período recortado (1899-1949). A seleção de edições analisadas passou pelo uso das palavras-chave "remo", "*rowing*", "regatas", "rio Tietê", "Tietê", "natação", "Travessia de São Paulo a nado". O jornal *A Gazeta* conta com edições que vão desde 1914 até 1933, e foi submetido ao mesmo processo de análise, pautado nas mesmas palavras-chave. Especificamente, uma reportagem do jornal *O Estado de S. Paulo* foi utilizada na elaboração do texto. Ressalta-se que essa reportagem foi localizada a partir de uma menção no *Correio Paulistano*.

Os jornais foram selecionados porque constituíram importantes elementos no processo de urbanização de São Paulo, divulgando discursos relacionados àquilo que se considerava moderno (Cruz, Peixoto, 2007). A imprensa, em seus mais diversos formatos, foi uma força ativa na história do capitalismo e das cidades, e não mera coadjuvante. Isso significa que, além de ser influenciada por inovações do mundo moderno, ela também produziu novos signos e discursos (Cruz, Peixoto, 2007). Para Cruz (2000), a expansão da imprensa periódica foi a faceta cultural mais importante do processo de formação e transformação da vida urbana em São Paulo.

Entre os discursos produzidos e difundidos pela imprensa paulistana, encontrava-se o esporte. Mundo afora, os jornais são importantes fontes de pesquisa para o estudo da história das práticas esportivas e do cotidiano dos clubes, e em São Paulo não foi diferente (Góis Junior, Lódola, Dyreson, 2016). Vigarello (2008), ao explorar o contexto francês, aponta que o mercado jornalístico foi um importante elemento de difusão dos esportes, associando a tais práticas mensagens e propagandas que lhes davam uma nova roupagem, conectando-as à saúde e às boas práticas sociais. Como retorno, os esportes movimentavam cada vez mais esse mercado, já que se configuravam como assunto de interesse da população (Sevcenko, 2000). Assim, a aliança entre os discursos modernos e a centralidade das práticas esportivas nos jornais paulistanos do início do século XX tornam importante essa fonte de pesquisa sobre os discursos produzidos pelos clubes, pelas federações e autoridades esportivas no período delimitado pelo presente artigo.

Nos jornais selecionados para essa investigação, diversas reportagens foram publicadas a respeito dos esportes aquáticos e dos clubes de remo localizados às margens do Tietê. O *Correio Paulistano* contava, desde 1904, com uma seção específica intitulada "Rowing", em que narrava as ações esportivas ocorridas nos clubes de regatas. Nela, divulgavam-se provas, atividades festivas, festivais internos, questões relativas à regulamentação das práticas e diretrizes das federações. Grande parte dos termos pesquisados foram encontrados nessa seção, o que associa as narrativas sobre o rio Tietê às práticas esportivas aquáticas promovidas.

*A Gazeta* foi um dos mais importantes expoentes das práticas esportivas na cidade de São Paulo. Com projeto editorial arrojado, o periódico pretendia-se moderno, destinado a um centro urbano que, já naquele momento, "não podia parar" (Toledo, 2012, p.54). O esporte foi um dos elementos associados aos benefícios da vida urbana. Embora não contasse, na primeira década do século XX, com uma seção específica voltada para as narrativas dos esportes, paulatinamente esse tema se incorporou às seções do jornal, até a criação, em

1928, de um caderno esportivo como suplemento semanal e, duas décadas mais tarde, a criação d'*A Gazeta Esportiva* (Toledo, 2012). Em relação aos temas aquáticos, *A Gazeta* narrava com frequência o cotidiano dos clubes esportivos da cidade nas seções destinadas aos esportes. Por intermédio de seu editor na época, Cásper Líbero, esse jornal também se constituiu como um dos expoentes das práticas esportivas no rio Tietê, ao promover a Travessia de São Paulo a nado entre 1932 e 1944.[1]

As revistas produzidas pelos clubes também se estabeleceram como importante meio de divulgação dessas associações nas décadas analisadas. Era por meio delas que a direção dos clubes se comunicava com os demais membros, compartilhando leis, normas sociais, regras, condutas morais. Os benefícios das práticas esportivas, principalmente do remo e da natação, eram constantemente retomados. Neste artigo, buscaram-se opiniões e impressões a respeito do rio Tietê, tanto no que dizia respeito ao cotidiano do clube quanto a sua relação com as práticas esportivas, divulgadas em suas páginas. Com base em tais fontes, investigaram-se as transformações na interpretação dos clubes e da imprensa esportiva a respeito do rio nas décadas analisadas.

## O rio Tietê perante as novas interpretações sobre a natureza

Na última década do século XIX, o rio Tietê era um aliado imprescindível do crescimento da cidade. O estado de São Paulo detinha, já em 1885, mais de 40% da produção nacional de café, principal artigo exportado pelo Brasil (Queiroz, 2004). A riqueza proporcionada por esse produto transformou as dinâmicas da cidade, impulsionando a urbanização. Instalaram-se ferrovias para o escoamento do grão, bancos, companhias de energia elétrica e outras empresas de bens e consumo (Saes, 2004). Ao mesmo tempo, a população da cidade aumentava de forma exponencial, principalmente pela chegada de imigrantes. De acordo com Hall (2004), São Paulo foi, entre o final do século XIX e início do XX, uma das maiores cidades de imigração do mundo. Entre 1829 e 1949 chegaram mais de 2,5 milhões de imigrantes ao estado. Em 1934, o contingente de imigrantes ou descendentes diretos na cidade era mais de 1/3 da população. São Paulo caminhava para se tornar, já na primeira metade do século XX, a maior cidade do Brasil e uma das maiores do mundo. Sendo assim, é possível afirmar que São Paulo seguia rumos similares aos de espaços urbanos já estabelecidos na Europa, adquirindo os mesmos traços de "cidade moderna" que marcavam o que Bresciani (1984-1985) denomina o "momento culminante" de um complexo processo de perdas em relação às representações do tempo, aos sistemas de trabalho e ao *habitat* tradicional, substituindo-os por um tempo linear, controlado e que contribui para que a cidade moderna se constitua como o "espaço por excelência da transformação ... ela representa a expressão maior do domínio da natureza pelo homem e das condições artificiais (fabricadas) de vida" (p.39).

O rio foi imprescindível para as transformações que ocorreram na cidade (Nóbrega, 1978; Jorge, 2006; Zanirato, 2011). Além dos elementos básicos para a infraestrutura, como pedras, areia e água, ele forneceu também energia elétrica, associado a seus afluentes. Em 1899, chegou em São Paulo a Tramway, Light and Power Company, empresa canadense-americana que atuava no setor de energia. Em 1901, essa companhia inaugurou uma usina nos arredores da cidade, e em 1908 construiu uma represa que regulava a produção de energia com o acúmulo das águas da chuva (Jorge, 2006). Além do ramo da energia, a empresa atuou também como agente imobiliário na região do Tietê, aumentando a especulação de valores das terras e interferindo, mesmo que indiretamente, no povoamento das margens do rio (Santos, 2011), assim como na intensidade das enchentes nos períodos chuvosos (Sevcenko, 2000; Jorge, 2006).

Por volta de 1890, essas novas dinâmicas urbanas já haviam tomado as margens do Tietê, especialmente na zona Norte da cidade. Cada vez mais moradores eram atraídos para a região, e inúmeros bairros surgiram nesse período. Isso significa que a fauna e a flora começavam a sofrer impactos da crescente urbanização. Além disso, a poluição das águas já preocupava os cientistas, que a associavam às doenças e às epidemias que assolavam cidades brasileiras no período (Marques, 1995; Chalhoub, 2001). A identificação da saúde como um problema central no desenvolvimento do país foi um dos motes da Primeira República brasileira, o que a tornou objeto de intervenção a partir de diferentes olhares.

Dessa forma, uma perspectiva de saúde relacionada a uma ideologia altamente moralizante foi propagada no período, tendo São Paulo como carro-chefe de novas políticas públicas, especialmente aquelas voltadas para o saneamento (Hochman, 2013). Os serviços de saúde da cidade de São Paulo, que contavam com combatentes audazes nessa cruzada social e moral, atribuíam ao mau cheio e à sujeira dos rios ou de outros fluidos a origem de diferentes doenças e malefícios (Mastromauro, 2010; Jorge, 2012; Mota, 2018).

O rio Tietê, portanto, entrou no escopo de preocupações médicas relativas à saúde pública em São Paulo. Artigos publicados em jornais da época procuraram alertar a população para as questões ligadas à saúde e ao contato com as águas dos rios. Em 1898, *O Estado de S. Paulo* publicou uma reportagem com análises dos exames microbiológicos realizados por médicos no trecho do rio Tietê que passava pela capital paulista. A notícia evidenciava que os dois grandes problemas do rio eram o percurso sinuoso, que fazia a água correr com menor velocidade e se acumular nas várzeas, e a grande quantidade de habitantes que começava a se aglomerar em suas margens, fato que tornava a água "sujeita a contaminações de toda a espécie" (A água..., 17 out. 1898, p.1).

A matéria jornalística ainda informou ter sido encontrada alta concentração do *Bacillus coli* (atualmente *Escherichia coli*), agente causador da cólera, bem como vetores de outras doenças, como a febre tifoide. Segundo o periódico, os surtos de doenças ocorridas em diversas cidades brasileiras eram advindos do consumo de água contaminada, e, no caso do Tietê, "nem mesmo pela filtração se encontrará um corretivo eficaz" (A água..., 17 out. 1898, p.1). O resultado do relatório dos médicos publicado no jornal paulistano atestava que a água do rio não deveria ser lançada ao consumo público. Jorge (2006) analisou os relatórios públicos do início do século XX, que também afirmavam que a situação sanitária do rio era preocupante, especialmente por conta da população que se acumulava em suas margens e do lixo que era carregado em maior volume nos períodos de chuva. Para os médicos e intendentes da cidade, seria necessário retificá-lo para que as águas e a sujeira deixassem de se acumular.

Alheia a tais problemáticas discutidas pelos médicos se encontrava parte da população paulistana. Com a aproximação de bairros populares das margens do rio, suas águas começaram a se tornar cada vez mais locais de divertimento. Jorge (2006) argumenta que paulatinamente a região do Tietê, especialmente na região da zona norte da cidade, passou a ganhar novos moradores, fosse pela especulação que se fazia em relação aos terrenos da várzea, fosse pelas distintas mobilizações urbanas que se passavam na cidade, decorrentes de processos de urbanização e transformação geográfica (Bresciani, 1984-1985). O memorialista Ernani Silva Bruno (1954) recorda momentos em que ele e seus amigos nadavam nas águas do Tietê. Entretanto, para isso era necessário fugir dos policiais, pois o poder público da época não autorizava o uso das águas para banhos e/ou exercícios físicos. Bruno (1954) afirma que a década de 1880 foi um período em que algumas leis foram promulgadas na tentativa de impedir a natação. As alegações eram o atentado ao pudor pelos nadadores nus e os perigos de afogamento. O nado, nesse momento, não era ainda considerado um esporte saudável, mas um jogo, considerado pouco sério e de má fama.[2]

Os afogamentos também eram problemas constantes na cidade. De acordo com as reportagens dos jornais, a população reclamava da falta de controle nas margens dos rios, descaso também apontado por Sevcenko (2000) no que diz respeito à atenção às famílias que viviam nas margens dos rios, especialmente durante os períodos de enchentes. Ao mesmo tempo, as autoridades atribuíam as tragédias aos banhistas, que adentravam as águas sem saber nadar. Tais reportagens suplicavam que o poder público aumentasse a vigilância e o policiamento nos trechos mais utilizados do rio, a fim de evitar novos afogamentos (Empreitada..., 23 mar. 1914; Menor..., 17 fev. 1907). Nesse contexto, é possível perceber que, ao mesmo tempo que a população incluía, de forma consciente ou não, o Tietê no rol de espaços disponíveis para os divertimentos, o poder público, quando tomava providências em relação aos arredores dos rios, promulgava leis que procuravam distanciar os habitantes das águas, restringindo seus usos.

Entretanto, uma nova concepção sobre a natureza chegava a São Paulo nesse período. No Brasil, um ideário de vida ao ar livre, que elegia determinadas características da natureza como provedoras de educação, cura e regeneração, instituiu-se entre fins do século XIX e início do XX (Segawa, 1996; Dias, 2010; Soares, 2016; Soares, Santos Neto, 2018). São Paulo e outros grandes centros urbanos brasileiros se valeram de discursos que elegiam a vida em meio à natureza como moralmente mais benéfica que aquela na cidade (Soares, 2016; Soares, Santos Neto, 2018; Silva, Quitzau, Soares, 2018; Dalben, Silva, 2020). Esses discursos, difundidos por médicos, higienistas, sanitaristas, arquitetos e professores, promoviam estratégias de aproximação e aproveitamento da natureza, considerando-a meio profícuo de educação e saúde da população.

Uma nova relação com a natureza, que se estabelecia em um Brasil urbano em fins do século XIX e início do XX, era fruto de uma concepção embasada em retóricas que promoviam uma reconciliação entre a natureza e a humanidade. Esses discursos são fruto de uma nova ideia de natureza, que se instituiu ao longo de mais de três séculos por meio de ideias e conceitos que acabaram por reabilitar o mundo natural no Ocidente. O século XVIII foi o momento em que essa reconciliação entre natureza e humanidade se tornou mais evidente, e uma nova sensibilidade perante seus elementos se colocou em marcha. Dessa forma, o mundo natural passou a compor discursos educativos, curativos e regenerativos, associados a qualidades como generosidade e benevolência (Thomas, 1984; Corbin, 1988, 2001, 2013; Rauch, 1995; Sirost, 2009; Villaret, 2005).

Em São Paulo, que crescia e se urbanizava, novas concepções a respeito da natureza começavam a se difundir e pautar a criação de espaços públicos. Por exemplo, foram estabelecidos parques e jardins urbanos nas regiões centrais da cidade; houve também a fundação de escolas e parques ao ar livre, para o usufruto das crianças (Segawa, 1996; Ferreira, Wiggers, 2019; Dalben, 2009; Dalben, Danailof, 2009; Soares, Santos Neto, 2018).

As novas interpretações não vieram apenas da população em geral, mas principalmente das autoridades. Estabeleceu-se uma institucionalização da vida ao ar livre, com diversas ações do poder público em relação ao bom aproveitamento de parques, praças, rios, entre outros espaços (Soares, Santos Neto, 2018). Nesse ínterim, as práticas de natação, os desafios aquáticos, os saltos nas águas, que já eram realizados no Tietê, começam a ser ressignificados (Jorge, 2012; Medeiros, 2020, 2022).

Essa transformação de sentidos se estendeu ao Tietê, quando clubes esportivos se instalaram em suas margens. A partir de então, começaram a ser tecidas novas ligações entre a prática esportiva e a educação em meio à natureza. Para além das escolas, dos jardins e dos parques infantis, as práticas esportivas realizadas no rio Tietê passaram também a contar com adjetivações positivas que relacionavam a vida em meio à natureza e a saúde. O remo e a natação, principais práticas desenvolvidas pelos clubes em meio aquático, eram a maneira ideal de promover saúde, divertimentos e educação aos associados.

## Tietê: rio que cura e diverte

Foi na virada do século XIX que os primeiros clubes esportivos se instalaram nas margens do rio Tietê (Nicolini, 2001; Jorge, 2012; Almeida, 2013; Medeiros, 2020, 2022). No ano de 1899 foi criado o Clube Esperia, instituição fundada por imigrantes italianos que desejavam praticar o remo. Em 1902, em sua outra margem, instalou-se o Clube de Regatas São Paulo, constituído por brasileiros que também praticavam esse esporte. Alguns anos depois essa associação fechou, originando o Clube de Regatas Tietê (1907) e a Associação Atlética São Paulo (1914). Inúmeros outros clubes fixaram-se nas margens do rio nas décadas seguintes (Nicolini, 2001; Almeida, 2013).

Essas agremiações optaram por estabelecer suas sedes na região da Chácara da Floresta, região da Ponte Grande, importante reduto de divertimentos dos habitantes da cidade de São Paulo no início do século XX. Essa região foi, nas primeiras décadas dos anos 1900, local de difusão de diversas práticas da cultura física, e nela dispositivos esportivos se instalaram, como campo de futebol e velódromo. Com a instalação dos clubes de regatas a Chácara da Floresta passou a ser frequentada por grande quantidade de pessoas (Gambeta, 2014). Os espaços verdes existentes nos clubes, repletos de árvores e em contato com o rio, eram frequentemente associados a uma fuga do mundo urbano e à promoção do contato com a natureza. Configuravam-se como espaço de relaxamento das horas de trabalho e da fadiga do cotidiano. No Clube Esperia, as atividades nos finais de semana eram agregadas à possibilidade de um "repouso regenerador tão necessário para todos que ficam encerrados o dia todo, por uma semana inteira, em escritórios e oficinas" (Piqueniques..., fev. 1932, p.6).

O desenvolvimento dos esportes nessas associações aconteceu paralelamente à atribuição de características morais e educativas a essas práticas. De acordo com Bourdieu (2003), o esporte não surge como um fenômeno desinteressado, e, sim, com o objetivo de inculcar novos valores à juventude, como a liderança, a coragem e a energia. Vigarello (1995, 2002) aponta que o esporte legitima o lazer, atribuindo-lhe questões morais. Com claras intenções atreladas às práticas esportivas, os clubes passaram a promover a natação e o remo em suas sedes.

A natação era associada a todos os benefícios conhecidos dos exercícios físicos. Ela promovia a força, a moralidade, o desenvolvimento geral do organismo e dos sistemas respiratórios, simbolizando em um único esporte todas as considerações teóricas dos higienistas sobre as diferentes práticas corporais (Arnaud, 1987; Terret, 1994; Love, 2007; Vigarello, 2019). Além disso, associava-se a natação ao confronto com o meio ambiente, o contato com o ar puro e com as baixas temperaturas da água, todos esses elementos considerados salutares para o organismo.

Nos clubes de São Paulo, eram exatamente essas as características atribuídas à natação. Em sua revista, o Clube Esperia assinalou os benefícios do exercício: "A natação constitui o mais completo de todos os exercícios, sendo ao mesmo tempo higiênico, estético e utilitário; deve desenvolver a força muscular propriamente dita, tanto como a força de resistência e fazer adquirir, igualmente, a destreza e a energia moral" (Vantagens..., 1928, p.14).

A natação realizada nas águas do Tietê não se configurava apenas como um exercício físico; a ela era atribuída também a característica de ser um confronto com o meio ambiente. Nadar significava mais do que se manter sobre a água: significava sobretudo não ser levado pela correnteza e enfrentar os desafios existentes no caminho. O nadador era reconhecido como um desbravador: "O nadador é intrépido sem arrogância. Rompendo as águas vai graduando as forças para que o não traiam em esmorecimento de fadiga; educa o ânimo mantendo-o vígilo e sereno e, no maior perigo, conserva-se imperturbável, sem angústias que atordoam, sem terrores que entibiam" (Netto, 1928, p.1).

O remo também ganhou conotações positivas nas disposições dos clubes. A ele se atribuía a formação integral do ser humano, física, social ou mentalmente. No Rio de Janeiro o remo já havia adquirido, em fins do século XIX, características que o tornaram representante da moral burguesa esportiva, da educação pelo esporte e da modernização da sociedade. Ele era considerado o esporte da saúde, do desafio, do progresso, da limpeza e da beleza (Jesus, 1999; Lucena, 2001; Melo, 2001, 2021). Tais concepções, que chegaram primeiro à capital brasileira, foram apropriadas também por clubes paulistanos e de outras partes do Brasil, que compartilhavam sentidos comuns atribuídos ao remo.

Os clubes de São Paulo também estabeleciam tais paralelos entre o remo e o desenvolvimento integral dos praticantes. Esse esporte era considerado o mais completo, técnica e psicologicamente (O Valor..., 1928, p.12); tratava-se da "verdadeira aliança do cérebro com o físico" (Como se fazem..., 1940, p.3). Além disso, exigia que os atletas tivessem dedicação e constância nos treinamentos, para que fossem capazes de enfrentar os desafios do rio (p.3).

O esporte serviu ainda como chamariz de questões como higiene e regeneração da raça, discussões que estavam em voga na cidade no período (Dalben, Góis Junior, 2018). Nas décadas de 1910 e 1920, o então prefeito da cidade, Washington Luís, entusiasta dos esportes, apadrinhou diferentes competições esportivas, entre elas, uma regata disputada no rio Tietê

e que passaria a levar seu nome (Sevcenko, 2000, p.256). Sua intenção era incentivar o que o próprio político e futuro presidente do país chamou de “aperfeiçoamento das populações”.

Cabe destacar que nascia, no Brasil, em meados do século XIX, uma ciência médica, embasada pela instalação das primeiras escolas de medicina do país (Schwarcz, 1993). Um dos principais apelos à nova carreira era o combate às doenças e epidemias que se espalhavam pelos centros urbanos. Surgiu, desses esforços, a ideologia da higiene, pautada na ideia de que um dos principais requisitos para que a nação brasileira atingisse grandeza e prosperidade e caminhasse em direção à civilização era a solução dos problemas de saúde pública. Em São Paulo, a partir da década de 1890, houve a intensificação dos debates a respeito da relação entre as doenças infectocontagiosas, a crescente urbanização e o aumento demográfico, traduzido em ações executadas pelo poder público em relação aos espaços e à população (Costa, 1979; Luz, 1982, 2014; Rago, 1985; Schwarcz, 1993; Marques, 1994; Bertucci, 1997, 2004, 2005; Chalhoub, 2001).

Para Hochman (2013), o período da Primeira República foi marcante para as mudanças em relação às concepções de saúde e doença, pensadas como problemas identitários nacionais, que deveriam ser combatidos em favor do nacionalismo. Nesse contexto, os debates ocorridos em São Paulo, bem como as diferentes reformas planejadas, apontaram para soluções das problemáticas de epidemias de saúde, desde o interior até o litoral, passando pela capital. Essas soluções tiveram como uma das principais vertentes o saneamento público, o que envolvia o fluxo dos rios, o controle do lixo e as questões como a profilaxia e a higiene. Dessa forma, pode-se medir a potencialidade do movimento sanitário paulista pela construção de aparatos burocráticos, pela criação de leis específicas e pelo incentivo às investigações científicas sobre as doenças.

Esse contexto se relacionava também com as novas concepções de cidade, de urbanidade e de cidadãos, com as quais as novas ideias atribuídas ao esporte dialogavam. Para Góis Junior (2013), o esporte moderno, ao se instalar em São Paulo, sustentava um estilo de vida, um capital específico e interesses gerais que eram influenciados – e ao mesmo tempo influenciavam – as estruturas materiais de urbanização e de crescimento econômico. Dessa forma, foi possível o surgimento de novas interpretações relativas aos exercícios da natação e do remo, qualificados positivamente tanto pelos clubes quanto pelo poder público. Tornava-se cada vez mais clara a associação entre higiene, saúde e as práticas esportivas: “Se há enfermidades que nos assaltam a súbitas, são mais frequentes as que nos entram no corpo pelas abertas [*sic*] do nosso descuido. Dois são os meios principais que temos para defender-nos de tais inimigas...; um é a cultura física, outro é a higiene” (Netto, 1935, p.1).

Tais relações estabelecidas entre as novas concepções de saúde e higiene e os esportes interferiam também nas percepções sobre o rio Tietê – espaço privilegiado onde ocorriam. Ele deixou de ser um local proibido para a prática de exercícios e divertimentos e passou a ter seu uso incentivado. As boas adjetivações que os esportes aquáticos recebiam diziam respeito também às suas ligações com a saúde e ao revigoramento promovidos pelos esportes, ao contato com a natureza e ao enfrentamento das águas. O Tietê era o elemento da vida ao ar livre que possibilitava esse encontro.

Se, por um lado, o rio era considerado o lugar da natureza que permitia o desenvolvimento de tais práticas, por outro lado ele se configurava como um obstáculo para a transformação e especialização dessas práticas esportivas. Com curvas em excesso e variação no volume d'água ao longo do ano (Jorge, 2006) ficava evidente que sua geografia atrapalhava o desenvolvimento de esportes de acordo com as regras internacionais. As competições de remo eram atrapalhadas pela impossibilidade de criação de uma raia. Os certames de natação sofriam com a variação de volume d'água, o que impedia a contabilização de recordes.[3]

Quanto mais os clubes se aproximavam dos ideais esportivos, menos interessante se tornava a prática nos rios. Os atletas paulistanos, desde a primeira década do século XX, conforme aponta Medeiros (2021), disputavam torneios nacionais e internacionais, e os clubes perceberam seu mau desempenho em comparação ao de atletas de outros países ou estados (O remo..., 1935, p.27). Isso foi atribuído à falta de especificidade do local de treinamentos e disputas; ou seja, o rio Tietê passava a ser questionado como lugar ideal para a realização de proezas atléticas. Esse fator foi responsável pela mudança de interpretação dos clubes a respeito do rio. De parceiro inestimável, ele passou, pouco a pouco, a ser visto como inimigo da especialização esportiva.

## Tietê: o rio que atrapalha, contamina e adoece

Com a preocupação dos clubes em relação ao desempenho esportivo dos esportes aquáticos, novos espaços passaram a ser procurados para a realização de treinamentos e torneios esportivos. Na natação, piscinas começaram a se tornar indispensáveis, espelhadas naquelas utilizadas nas maiores competições internacionais.

De acordo com os jornais do período, a construção de piscinas era necessária, já que o Brasil desejava se tornar uma nação esportiva. A piscina não melhoraria apenas o treinamento dos nadadores, como também seria "imprescindível para obtenção dos recordes nacionais" (A questão..., 20 fev. 1925, p.3). Elas foram diretamente associadas à melhoria do desempenho e à quebra de marcas e recordes esportivos.

Com espaço fechado, águas calmas e claras, e geometria simples, as piscinas atendem a um projeto formal de exercício, ou seja, oferecem uma arquitetura a serviço da precisão. Mundo afora elas se tornaram o local preferencial das práticas de natação, quando paulatinamente as práticas em águas abertas foram excluídas dos catálogos federativos por não atender a uma estrita norma esportiva (Chartier, Vigarello, 1982; Terret, 1994; Johansson, 2008; Nzindukiyimana, O'Connor, 2013; Nicolas, 2021).

Além da perspectiva técnica promovida pela piscina, havia também uma questão higiênica envolvida em sua construção. Nelas, o acesso dos nadadores podia ser controlado. Aos usuários poderiam também ser exigidas duchas higiênicas antes da entrada na água (Triboux, 2007; Wiltse, 2007; Nzindukiyimana, O'Connor, 2013; Carreras, 2020; Nicolas, 2021). Além disso, as análises das águas eram feitas a qualquer momento, e, caso necessário, os clubes seriam responsáveis por alterar sua composição química. Nos Estados Unidos, em meados da década de 1890, houve a disseminação da ideia de que as piscinas eram causadoras de doenças infecciosas. Dessa forma, a mudança de regras higiênicas passou a ser implantada na tentativa de minimizar essas contaminações (Wiltse, 2007). No período aqui analisado, uma literatura a respeito da contaminação das águas de piscinas e sobre maneiras de prevenção já era bastante disseminada nos clubes.

Os clubes se ocupavam das discussões higiênicas sobre as águas das piscinas, publicando até mesmo teses médicas sobre o tema (Problema..., jun. 1932, p.5). Nessas teses, o ponto de comparação era o Tietê, considerado inapropriado para a utilização humana por conta das bactérias presentes em sua água. Assim, o nado na piscina, além de mais técnico, era considerado muito mais higiênico. Tendência similar pode ser observada em cidades como Barcelona, onde, em meados do século XX, foi possível observar um processo de ampliação na quantidade de piscinas internas e de clubes de natação, cujas finalidades estavam fortemente ligadas a intenções de intervenção urbanística relacionadas à higiene, assim como a preocupações relativas à higiene e saúde da população (Carreras, 2020).

A piscina do Clube de Regatas Tietê, construída em 1934, foi um importante marco nessa diferenciação higiênica em relação ao rio. Esse espaço foi abastecido, nos seus primeiros anos, pela água do próprio Tietê. Entretanto, o líquido passava por tratamento intensivo para que se tornasse adequado ao uso (Tratamento..., 3 jun. 1934, p.29). Ficava evidente a marca higiênica que as piscinas traziam em relação ao uso das águas.

Além das questões higiênicas, o rio também passou a ser associado a outros problemas que acometiam os clubes de regatas e/ou as disputas esportivas, como, por exemplo, as enchentes. Inúmeras foram as ocorrências de enchentes em São Paulo na primeira metade do século XX, momento de expansiva urbanização da cidade (Sevcenko, 2000; Jorge, 2011; Santos et al., 2014). Em geral, os resultados dessas enchentes foram catastróficos para a população. Os clubes de regatas também foram atingidos, e as suas críticas passaram a ser direcionadas ao “excesso de natureza” do rio.

O Clube Esperia fechou sua sede por mais de uma semana, depois de uma cheia do rio ocorrida em 1929. As disputas esportivas que seriam realizadas naquela semana também foram canceladas. Além disso, os atletas da entidade não puderam treinar para as competições futuras (A enchente..., 1929, p.1). Em 1931, a mesma situação se repetiu: “A enchente do rio Tietê atingiu este ano também a nossa sede que ficou com a maior parte de suas dependências alagadas. ... Tendo baixado o nível das águas todas as secções esportivas recomeçaram regularmente seus treinos” (A enchente..., 1931, p.15). Inúmeras outras variações de volume das águas foram responsáveis por alterações nos calendários das federações aquáticas (Remo, 1935, p.11).

Para os clubes e as federações, era urgente que esses problemas fossem reparados e atenuados, especialmente para a manutenção e o incremento das práticas esportivas. Uma das ideias em voga no período era a transformação do traçado do rio para a construção de raias para disputas de remo, como as existentes na cidade do Rio de Janeiro: “[Um] elemento essencial é a raia de regatas, que o nosso lendário rio Tietê somente poderá proporcionar aos *Clubs* de remo quando for retificado ou quando ela for construída especialmente para esse fim. O esporte do remo, apesar de ser praticado em nosso estado há mais de trinta anos, continua por aquele motivo na sua fase quase que primitiva” (Os paulistas..., 1935, p.5).

Assim, é possível perceber que, na interpretação das associações, o rio não comportava mais as práticas de natação e de remo. A necessidade de especificação dessas atividades, como a anotação de recordes, a melhoria de treinamentos e a demarcação de percursos, contrastava com a rebeldia das águas (Medeiros, 2020). Com a busca por outros espaços para as competições, como as piscinas e as represas, o rio deixava de ser o parceiro das práticas esportivas. De espaço ao ar livre responsável por promover higiene e saúde, ele passava, paulatinamente, a ser associado aos malefícios da urbanização.

De acordo com Jorge (2006, 2012), no início dos anos 1940, o Tietê era um rio cada vez mais degradado e perigoso para os habitantes da cidade. Quase nenhuma espécie animal vivia em suas águas nos trechos urbanos, e a mata ciliar havia sido destruída para dar lugar a prédios, casas e indústrias (Santos et al., 2014). O crescimento da cidade, aliado à falta de políticas de preservação ambiental, levou à completa degradação do rio no trecho da Ponte Grande, recorte geográfico dessa pesquisa.

Logo no início da década de 1940 as reportagens sobre o Tietê nos jornais paulistanos já apontavam essas mudanças. Em geral, conforme aponta Jorge (2012), o rio era considerado um grande problema de saúde pública. As questões esportivas eram mencionadas com pesar, e, para os periódicos, tratava-se de um dos maiores prejuízos da contaminação das águas:

> Os aspectos estético e esportivo devem também ser encarados neste apanhado de prejuízos resultantes da poluição das águas. Realmente, é pouco agradável o aspecto de um rio de águas escuras e em cuja superfície se distinguem detritos de toda a natureza ... É repugnante o mau cheiro de um rio nessas condições, e, sendo suas águas impróprias para os esportes aquáticos, priva-se a população ribeirinha de um dos mais sadios meios de educação física (O problema..., 9 jun. 1940, p.20).

Sem a associação com os esportes, e com águas cada vez mais poluídas, o Tietê deixou o *status* benevolente que havia alcançado nos primeiros anos do século XX e passou a constar no rol de problemas públicos da cidade. Foi na década de 1940 que Jorge (2006, 2012) identificou um momento de distanciamento da população do Tietê, com o abandono de suas margens. Deixava-se de utilizar os rios para os divertimentos, os passeios, as pescarias e para outros eventos sociais.

Foi nesse momento também que os clubes deixaram de utilizar o rio para a promoção de esportes e demais atividades associativas. As competições de natação, que já eram realizadas nas piscinas, passaram a ser oficialmente disputadas nas sedes dos clubes, por determinação da federação (Esperia..., 9 jan. 1939, p.13). As competições de remo passaram a ser realizadas no litoral do estado, na cidade de Santos, e/ou na represa de Santo Amaro que se localizava na cidade vizinha de mesmo nome. O marco final para essa relação entre o Tietê e esse esporte foi 1948, ano em que foi criada a raia de Jurubatuba, no caminho para o litoral. A partir de então, as competições oficiais foram transferidas para lá (Na raia..., 17 out. 1948, p.12).

As mudanças estruturais pelas quais o Tietê passou encerraram, definitivamente, as possibilidades de relação entre os clubes e as águas. Na década de 1940, foi colocado em ação o Plano de Avenidas, elaborado pelo então prefeito da cidade, Prestes Maia. Tais obras pretendiam resolver o problema das cheias do rio e iniciar um processo de retificação. As obras realizadas pelo prefeito Prestes Maia pretendiam alterar o leito do rio e tornar a região mais aproveitável economicamente. Embora alguns aspectos das obras tenham beneficiado a malha urbana, as alterações no traçado do rio mudaram, de uma vez por todas, a sua relação com a fauna e a flora local, bem como a possibilidade de realização de práticas esportivas (Carpintero, 2013). Tais remodelações demoraram anos e acabaram estreitando a largura do rio e diminuindo a sua profundidade. Tal fato acabou, de uma vez por todas, com as disputas esportivas nas águas do Tietê.

## Considerações finais

Este artigo teve como objetivo analisar as interpretações a respeito do rio Tietê e as transformações de discursos sobre ele nas primeiras quatro décadas do século XX, sobretudo naquilo que se relaciona às atividades esportivas. Conclui-se que houve uma mudança de interpretação dos clubes e da imprensa a respeito do rio, marcada temporalmente ao longo do período analisado.

Nas duas primeiras décadas do século XX, nas quais os esportes aquáticos se desenvolveram amplamente em suas águas, havia uma concordância sobre seus benefícios em prol da saúde e da educação. Os pressupostos da higiene, associados aos esportes aquáticos, eram tomados como importantes elementos na difusão dessas atividades. O rio, além de espaço geográfico em que tais práticas podiam ser realizadas, era a composição ideal com a natureza, com a aventura, e o enfrentamento de seus elementos constituía-se em algo crucial para as definições de uma educação relacionada à natureza no período analisado.

A partir da década de 1930 esse discurso se alterou. Os clubes começaram a notar a falta de especialização de seus nadadores e remadores que não treinavam em espaços especificamente criados para disputas esportivas. Assim, foi crescente a construção de piscinas nessa época, bem como as tentativas de realização de campeonatos de remo em outros locais. Pouco a pouco, o Tietê deixava de ser o espaço principal de competições e treinamentos esportivos. Toda a associação entre a saúde, a higiene e os esportes, construída nas décadas anteriores, deixou de acompanhar as impressões sobre o rio.

Nota-se que os discursos a respeito da poluição das águas do rio no trecho urbano já eram divulgados desde o final do século XIX. Entretanto, os clubes optaram por minimizar essa problemática, enquanto o Tietê era parceiro imprescindível à prática esportiva. A partir do momento em que outros espaços mais convenientes, como as piscinas e as represas, foram construídos, os discursos negativos a respeito do rio tomaram conta e reinaram absolutos nas publicações dos jornais e das revistas dos clubes.

Em estudos sobre o desenvolvimento esportivo da natação na França, Terret (1994) nota que as águas abertas não eram prejudiciais à higiene dos praticantes, e, sim, ao desenvolvimento esportivo da natação. Em São Paulo situação semelhante foi percebida: antes da total impossibilidade do uso do rio, os discursos que priorizavam as questões esportivas já tinham condenado o Tietê ao posto de transmissor de doenças.

Na década de 1940, esse processo de distanciamento se intensificou, e os últimos torneios de natação e regatas foram disputados. Os clubes se fecharam cada vez mais dentro de suas sedes e colocaram muros para separar-se das águas dos rios. O desfecho dessa relação se deu com a construção de avenidas marginais idealizadas por um plano urbanístico que separou fisicamente os rios e os clubes e retificou seus trechos urbanos. A partir dessa década, de acordo com Jorge (2006), o Tietê se tornou tão degradado que se transformou em uma ameaça a todos os seres vivos que se aproximavam de suas águas – condição que perdura até os dias atuais.

## NOTAS

[1] Embora a prova tenha sido estabelecida pelo jornal *São Paulo Esportivo* em 1924, o auge da competição se deu durante o patrocínio d'*A Gazeta*. Para outras informações sobre a Travessia, consultar Medeiros (2020).

[2] Questões semelhantes foram encontradas por Arnaud (1987), Terret (1994), Vigarello (2002, 2019) e Machemehl (dez. 2011) em estudos sobre as transformações da natação na França. Os autores identificam que, antes de a prática adquirir caráter de esporte, ou seja, quando ainda se tratava de algo popular, ela era malvista nas cidades analisadas, especialmente pelas autoridades. O nado "selvagem" não era tolerado, tanto pelo decoro quanto pela segurança dos banhistas.

[3] Nas provas da Travessia de São Paulo a nado (a maior prova de natação realizada no rio) os melhores recordes foram obtidos nos anos em que o rio estava mais volumoso (Medeiros, 2020).
